# Hepatitis E Virus RNA Detection from Hunted Wild Boars in Central Italy: an Epidemiological Investigation

**DOI:** 10.1007/s12560-023-09554-3

**Published:** 2023-04-07

**Authors:** Gianluigi Ferri, Giorgia Giantomassi, Andrea Piccinini, Alberto Olivastri, Alberto Vergara

**Affiliations:** 1 Department of Veterinary Medicine, Specialization School in Food Inspection “G. Tiecco”, University of Teramo, Piano d’Accio, Strada Porvinciale 18, 64100 Teramo, Italy; 2 Veterinary Practitioner, Ascoli Piceno, Italy; 3 SIAOA Veterinary Public Service, Ascoli Piceno, Italy

**Keywords:** HEV, Viral RNA detection, Liver and muscle tissues, Molecular biology, One health

## Abstract

Every year, foodborne pathogens, including the hepatitis E virus (HEV), cause thousands of infections in different continents. Final consumers become infected through the ingestion of contaminated animal origin foodstuffs. Generally, in industrialized countries, HEV genotype 3 is involved in sporadic outbreaks. Infections have been described, in Europe and Japan as consequence of pork products and contaminated wild boar’s primary or processed products (liver and muscle tissues) consumption. In Central Italy, hunting activities are largely practiced. In these small and rural communities, game meat and liver are ingested by hunters’ families or at local and traditional restaurants. Therefore, these food chains can be considered critical HEV reservoirs. In this study, 506 liver and diaphragm tissues were collected from hunted wild boars in the Southern Marche region (Central Italy) and were screened for HEV RNA detection. From the 10.87% of liver and 2.76% of muscle samples, HEV3 subtype c was discovered. The observed prevalence values resulted in line with previous investigations performed in other Central Italian regions, but higher than Northern ones (3.7% and 1.9% from liver tissue). Therefore, the obtained epidemiological data highlighted the wide occurrence of HEV RNA circulation in a low-investigated area. Basing on results, a One-health approach was adopted due to the sanitary relevance of this Public Health concern.

## Introduction

The World Health Organization estimated that about 20 million (with 3.3 million symptomatic patients) hepatitis E virus (HEV) infections occur every year (WHO, 2018). Between 2005 and 2015, HEV caused more than 21,000 clinical cases in Europe. Most of them were circumscribed to specific territories (i.e., Germany, France, Spain, and Italy) (Aspinall et al., 2017).

According to the HEV taxonomic classification, it belongs to the *Hepeviridae* Family, Subfamily *Orthohepevirinae*, *Paslahepevirus balayani* species, and it is a quasi-enveloped positive sense RNA virus (Purdy et al., 2017). The viral genome is characterized by three Open Reading Frames (ORF): ORF1 encodes nonstructural proteins, ORF2 encodes a capsid protein, and ORF3 a small protein (Koonin et al., 1992). Full-length genome analysis permitted the classification of HEV strains into eight genotypes (from HEV1 to HEV8). Genotype 1 and 2 have been widely detected in low-income countries and only in human patients. Secondly to the outbreaks’ occurrence, the epidemiological investigations have demonstrated that contaminated water ingestion was the main HEV source (Aspinall et al., 2017). Concerning HEV3 and HEV4, many studies observed that these two genotypes are responsible for sporadic infections in industrialized countries due to the consumption of contaminated food matrices (Smith et al., 2020; SEIEVA, 2022). The full genome analysis, supported by the bio-informatic assays, revealed the HEV3 and HEV4 involvement as zoonotic genotypes which generally caused autochthonous sporadic cases in Europe, the USA, and Japan (Salines et al., 2017). The identified genomic sequences (belonging to HEV3 and HEV4), isolated from foodstuffs of animal origin and human species, result closely related to each other. This consideration supported zoonotic transmission (Colson et al., 2010). HEV genotypes can be classified into various subtypes which differ in virulence and morbidity factors (Smith et al., 2020; Yadav & Kenney, 2021; Wißing et al., 2021). Furthermore, the HEV-3 gropus1 (which includes subtypes 3e, f, and g) caused a more consistent number of infections than the group 2 (3a, b, c, h, i) resulting identical in patients and leftover food consumed by them (Schemmerer et al., 2022). The association of HEV subtypes virulence to the related severity of caused symptoms has been purposed as a possible scientific hypothesis, but it needs more detailed studies to demonstrate these correlations (Abravanel et al., 2020; Hoa et al., 2021; Schemmerer et al., 2022). In Europe, Asia, and Northern American continents, HEV3 subtypes from 3a to 3 m and HEV4 from 4a to 4 g were widely detected (Smith et al., 2020) with special regard to the 3f (Abravanel et al., 2020; Schemmerer et al., 2022). Focusing on the European scientific literature, HEV3c, 3e, and 3f subtypes were mainly discovered, with special regard to the domestic pig (*Sus scrofa domesticus*) which represents the main HEV reservoir in the so-called “*domestic viral life-cycle*” (Di Bartolo et al., 2017; Ivanova et al., 2015), and wild boars (*Sus scrofa*) resulted to be involved in the “*wild one*.” Furthermore, due to the increasing anthropization and urbanization processes, the environmental sharing with wild animal species (wild boars and ruminants) provides epidemiological and ecological conditions for possible cross-species infections (Anheyer-Behmenburg et al., 2017; Casares-Jimenez et al., 2021; Chandra et al., 2008; Wu et al., 2022). Basing on the viral trophism for hepatocytes, and secondly to the viremia step, and myosatellite cells, the biomolecular assays on these mentioned tissues result necessary for further public sanitary considerations (Aspinall et al., 2017). Therefore, wild boar sera, liver, meat, and relative processed products were screened in many Italian regions: in North-central Italy 14.3% of liver samples were positive (Martinelli et al., 2015), North-western Italy (3.7% positive liver samples) (Caruso et al., 2015), Central Italy (16.3% positive liver samples) (Di Pasquale et al., 2019), Southern Italy: 13.7% positive liver samples (Aprea et al., 2018), 11.4% positive liver samples (Montone et al., 2019), and 10.2% ones (De Sabato et al., 2020a, 2020b). These studies mostly identified the following subtypes 3e, 3f, and 3c. However, few research groups examined the HEV RNA simultaneous detection in liver and diaphragm tissues sampled from a single animal highlighting average viral RNA detections of 10.0% (95% CI: 7.0–14.0%) from liver and 3.0% (95% CI: 0.04–10.0%) from muscle tissues (De Sabato et al., 2020a, 2020b; Fanelli et al., 2021). Therefore, the wide HEV persistence in hepatocytes and the limited detection in muscle tissues still represents a consistent concern for consumers due to the possible infections related to contaminated food consumption. More in detail, in these geographical areas, wild boar processed products (liver sausages, salami, etc.) are largely consumed avoiding the cooking process. From a One-health perspective, the human hematology groups, located in Marche region, were involved in the serological national screenings for anti-HEV IgG detection. This region was involved for the high-risk factor concerning HEV infection due to the wide consumption of hunted wild boar fresh or processed products as local traditional cooking culture. For this reason, all blood donors were part of this surveillance program and results showed a prevalence of 11.9% (CI 95%: 6.6–20.5%) of anti-HEV IgG, and more specifically, 13.6% (CI 95%: 9.3–19.3%) from females and 11.2% (CI 95%: 5.5–21.5%) from males (Spada et al., 2022).

This study aimed to screen the HEV RNA detection in 506 liver and diaphragm samples collected from hunted wild boars during the season 2020/2021 in the Southern Marche region (Central Italy, province of Ascoli Piceno). Following the previous epidemiological investigations [5,12% liver specimens resulted positive for HEV RNA detection (Ferri et al., 2022a) and 5.45% (Ferri et al., 2022b)] performed in the same province, the obtained results wanted to provide original data regarding HEV circulation highlighting possible changes regarding genotype or subtypes diffusion, after one year.

## Materials and Methods

### Sample Collection

During the hunting season 2020/2021 (from October 2020 to January 2021), a total of 506 wild boars (*Sus scrofa*) were collected. All of them were hunted in the Ascoli Piceno province, Marche region (Central Italy). The screened area was characterized by many environmental and cultural aspects: wide hunted designed zones (10,251 ha), high wild boars’ densities (3.5/100 ha) (Regional Marche Hunting Report, 2020), environmental sharing between domestic and wild animal species (including also wild ruminants), the persistence of a rooted hunting tradition and consumption of related primary or processed foodstuffs. Small and traditional swine farms, located in the hunting areas, are characterized by low anthropization levels, wide agricultural fields (located in the hunting zones) which are fertilized using pig manures. From each animal liver and diaphragm tissues were screened. All subjects were registered reporting the following information: sex, age (morphometric estimation based on extent of tooth eruption), place of death, and weight (See Table 1).

**Table 1:** Classification of screened animals based on sex and weight

	Age & weight categories
Males	
252	J* → 6 P*→ 111 A* → 135
Females	
254	J* → 2 P* → 115 A* → 137

*J = juvenile (weight < 15 kg and estimated age between 0 and 12 months)

*P = puberal (15 kg < weight < 40 kg, and estimated age between 13 and 24 months)

*A = adult (weight > 40 kg and estimated age between 24 and 48 months)

Therefore, the studied population was composed of 49.80% female (95% CI: 45.45–54.15%) and 50.19% (95% CI: 45.84–54.54%) of male subjects. More specifically, in both gender groups, adults represented the 53.57% (95% CI: 47.47–59.67%) and puberal the 45.63% (95% CI: 39.53–51.73%). Juveniles were lowly represented due to the selective hunting criteria introduced by the Regional Marche Law No. 3/2012, which aims to preserve the eco-sustainability of certain wild ecosystems. In accordance with the European Regulations (EU Reg.), each animal received *ante* and *post mortem* examinations respectively performed by hunters (in agreement with the EU Reg. No. 853/2004) and by veterinary sanitary authorities (EU Reg. No. 625/2017) and Regional Marche Law No. 3/2012. All hunted animals were also tested for *Trichinella* spp. detection (EU Reg. No. 1375/2015). Secondary to the gross pathology examination (as indicated in the above-mentioned European and National laws), livers and muscles (diaphragm and tongue) were sampled (15–20 cm^3^) at the slaughterhouse in Ascoli Piceno. After collection, all specimens were transported under refrigerated conditions to the laboratories and stored at–80 °C until processing and molecular screenings.

### Sample’s Processing and RNA Extraction

Five grams of liver and muscle tissue were singularly and aseptically collected from the screened animals. Each aliquot was suspended in 10 ml of sterile phosphate buffered saline (PBS, pH 7.2, PAN™ Biotech GmbH, Aidenbach, Germany). All samples were individually homogenized, using the T18 digital Ultra-Turrax® (Staufen, Germany), for 1 min. Each specimen was centrifugated at 4,000 g for 20 min at 4 °C. The supernatant was filtered, divided into aliquots with a final volume of 3 mL, and stored at − 80 °C until nested PCR assays, as reported by Szabo et al. (2015). The following step was represented by the nucleic acid extractions. RNA pellets were obtained following the TRIzol LS (Invitrogen, Ltd, Paisley, UK) procedure and were resuspended gaining a final volume of 50 μL using RNase free water (Invitrogen UltraPure DNase/RNase-Free Distilled Water, ThermoFisher™, Waltham, MA USA).

### Molecular Biology: Reverse Transcription PCR (RT-PCR) for HEV Amplification

In this study, consensus primer sets were used as specific targets for sequences belonging to the ORF1, as reported by Johne et al. (2010) and ORF2 (Wang et al., 1999) genes. All samples (liver and muscle tissues) were both analyzed using the above-mentioned couples of primers. More detailed information are schematically reported in the Table 2.

**Table 2:** Oligonucleotide sequences, amplicon sizes, and references concerning the used primers for biomolecular screenings

Genes	Oligonucleotides	Sequences (5 ‘-3 ‘)	Amplicon sizes	References
ORF1	HEV-cs*	TCGCGCATCACMTTYTTCCARAA	470 bp	(Johne et al., 2010)
	HEV-cas	GCCATGTTCCAGACDGTRTTCCA		
	HEV-csn**	TGTGCTCTGTTGGCCCNTGGTTYG	333 bp	
	HEV-casn	CCAGGCTCACCRGARTGYTTCTTCCA		
ORF2	HEV-ORF2con-s1*	GACAGAATTRATTTCGTCGGCTGG	197 bp	(Wang et al., 1999)
	HEV-ORF2con-a1	CTTGTTCRTGYTGGTTRTCATAATC		
	HEV-ORF2con-s2**	GTYGTCTCRGCCAATGGCGAGC	145 bp	
	HEV-ORF2con-a2	GTTCRTGYTGGTTRTCATAATCCTG		

*RT-PCR assay; **Nested PCR

D = A, G, T; M = A or C; N = A, C, G, T; R = A or G; Y = C or T

The Reverse Transcription-PCR (first reaction) assay, for both ORF-1 (HEV-cs and HEV-cas) and ORF-2 (HEV-ORF2con-s1 and HEV-ORF2con-a1) primers, was performed using the Qiagen® OneStep RT-PCR Kit (Hilden, Germany).Nested PCR (second reaction) (ORF-1 primers: HEV-csn and HEV-casn; ORF-2 primers: HEV-ORF2con-s2 and HEV-ORF2con-a2) was realized with the Green Master Mix Promega® (Madison, WI, USA), according to the manufacturer’s instructions. Both reactions were performed in a total volume of 25 µL. Thermocycler was set following references indications (Johne et al., 2010; Wang et al., 1999). After all PCR assays, amplicons were loaded on the agarose gel at different concentrations (1.5% or 2.0% depending on their sizes), and the positive detected bands were compared to specific DNA ladders (50 bp and 100 bp DNA Ladder, Genetics, FastGene®). The nested PCR products 333 bp (ORF1) and 145-bp (ORF2) were purified using the Qiagen QIAquick® PCR Purification Kit (Hilden, Germany), and were sequenced, as described by previous studies (Aprea et al., 2018; Di Bartolo et al., 2017). HEV subtype identification was based on the alignment into the HEV-Typing tool (De Sabato et al., 2020a, 2020b) (https://www.rivm.nl/mpf/typingtool/hev/). A total of 69 sequences (55 from liver and 14 from diaphragm samples) were analyzed through the BLASTN system (https://blast.ncbi.nlm.nih.gov/Blast.cgi, accessed on 22 June 2022) to evaluate the nucleotide similarities. These sequences were successively deposited (excluding the identical ones) on the GenBank database (https://www.ncbi.nlm.nih.gov/genbank/).

### Statistical Analysis

For all calculated percentages, when applicable, the confidential intervals (CI: 95%) were defined. The chi-square statistic value (associated to the Yate’s correction) calculation was performed using the XLSTAT 2014 software® (Renmond, Washington, DC, USA) considering significant the findings with final *p*-value < 0.05. The Pearson correlation coefficient analysis was calculated between two parameters.

## Results

**Fig. 1:**
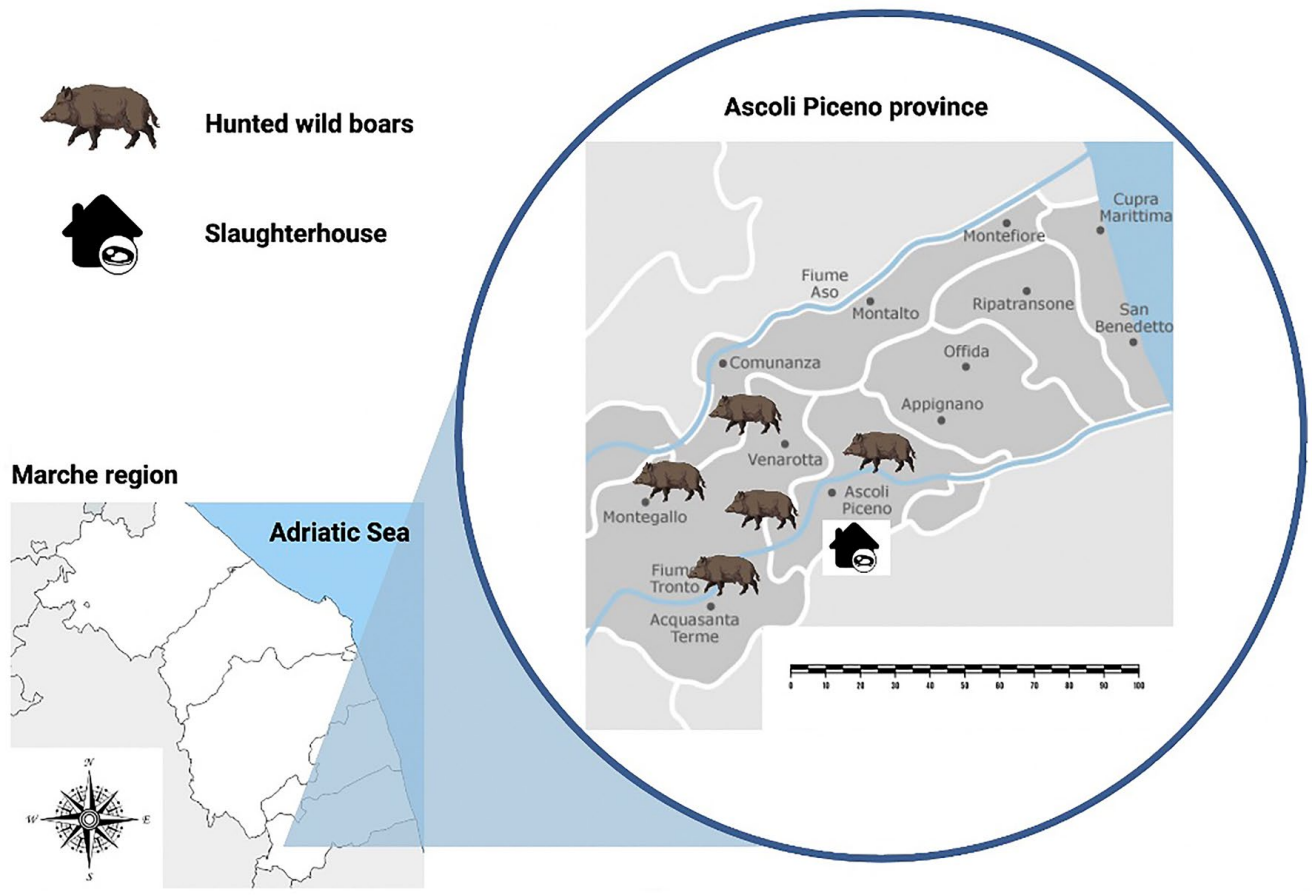
Hunted wild boars’ distributions among Ascoli Piceno province, Central Italy

In the hunting season 2020/2021, 506 livers and diaphragms were sampled from hunted wild boars (*Sus scrofa*) in the province of Ascoli Piceno (Marche region) in Central Italy. Basing on the territorial screened surface (10,251 ha), as previously indicated (Materials and Methods section), it was observed, that between positions, where positive animals were discovered, there was an average distance from 8 to 15 km, as illustrated in Fig. 1.

HEV RNA detection was observed in 55/506 of screened subjects which represented 10.87% (95% CI: 8.16–13.58%) of liver samples; 14/506 (2.76%–95% CI: 1.34–4.18%) of diaphragm specimens resulted positive. The ORF1 and ORF2 nucleotide sequences were both amplified from all positive animals. Basing on the gender factor, 38/55 (69.09%–95% CI: 56.68–81.30%) of positive animals were female and, more in detail, 23/38 (60.52%–95% CI: 44.98–76.06%) were adult and 15/38 (39.47%–95% CI: 23.93–55.01%) were puberal ones. Positive male subjects were 17/55 (30.90%–95% CI: 18.69–43.11%) of which 10/17 (58.82%–95% CI: 35.43–82.21%) were adults, and 7/17 (41.17%–95% CI: 17.78–64.56%) were puberal. Diaphragmatic HEV detection was always correlated to the respective hepatic one. Conversely to the two previous epidemiological investigations, performed in the hunting season 2019/2020, after one year, it was observed a significant difference (chi-square value χ2: 21.5684 with a *p*-value < 0.0001) between positive animals: 5.12% (Ferri et al., 2022a) and 5.45% (Ferri et al., 2022b) with the 10.87% discovered by this study.

More in detail, 10/14 of male positive muscle tissues represented the 71.43% (95% CI: 47.77–95.09%) and females were 4/14 being the 28.57% (95% CI: 4.91–52.23%), as schematically represented in the following Table 3.

**Table 3:** Sex-related organs HEV RNA positivity

Sampled organs		Positive livers	Sex-related livers’ positivity		Positive diaphragms	Sex-related diaphragms’ positivity	
Liver	Diaphragm		Female	Male		Female	Male
506	506	55	38 (69.09%)	17 (30.90%)	14	4 (28.57%)	10 (71.43%)

However, none of the animals were positive in muscles only. Sequences were published on the GenBank platform (https://www.ncbi.nlm.nih.gov/genbank/, accessed on 01 July 2022), and registered with specific accession numbers: MN20202101, MN20202102, MN20202103, MN20202104 for ORF1 fragment (333 bp), and MN2020210201, MN2020210208, MN2020210212 for ORF2 fragment (145 bp). All positive samples, 55 livers and 14 muscles, were sequenced in both ORF1 and ORF2. More in detail, the BLASTN results showed 98.00% nucleotide identity of 37/55 sequences (67.27%–95% CI: 54.87–79.67% from positive livers) of this study with HEV3 subtype c identified from wild boar livers and diaphragm in the same area in Marche region (GenBank accession number: ON364349.1) one year before (Ferri et al., 2022b). The HEV-Net Typing tool confirmed the subtype attribution to the 3c. Finally, 17/55 (30.90%–95% CI: 18.50–43.30%) of sequences obtained from livers, and 14/14 from diaphragm specimens presented 99.00% of nucleotide identity with the same above-mentioned genotype 3c with special regard to the GenBank registered sequence ON364350.1 which was also detected in the previous year. Only 1/55 of positive liver samples (registered as MN2020210208), exclusively for the ORF1 fragment, was 100.00% identical to the 3c registered with the following GenBank accession number: LC176493.1 by Miura et al. (2017) discovered from human sera. The registered sequences were selected basing on their own nucleotide similarities and on the GenBank ones. Starting from the ORF1 sequenced fragments, 37/55 (67.27%–95% CI: 54.87–79.67%) were identical each other (to the registered one with the accession number MN20202102). A group of 10/55 (18.18%–95% CI: 5.78–30.58%) of sequences (from positive livers) and 8/55 (14.54%–95% CI: 2.14–26.94%) were 98.00% identical and registered as MN20202103. One out of fifty-five sequences was registered as MN20202101 presenting 5% of nucleotide differences if compared to the others mentioned. Finally, the positive diaphragm sequences to the ORF1 fragment were identical and registered as MN20202104 (as illustrated in the Table 4).

**Table 4:** Geographical localization: hunting areas & GenBank registered positive animals

ID	Positive subjects	Positive tissues	HEV target genes	Localization
MN20202101	AF	LV	ORF1	Comunanza (AP): 42.57 N; 13.24 E
MN20202102	AM	LV		Ripatransone (AP): 42.59 N; 13.45 E
MN20202103	AF	LV		Mozzano (AP): 43.58 N; 10.32 E
MN20202104	AF	DG		Mozzano (AP): 43.58 N; 10.32 E
MN2020210201	AF	LV	ORF2	Roccafluvione (AP): 42.51 N; 13.28 E
MN2020210208	AF	LV		Comunanza (AP): 42.57 N; 13.24 E
MN20202101212	AF	DG		Rotella (AP): 42.57 N; 13.33 E

*ID* identification on GenBank, *A* adult subject, *M* male, *F* female, *LV* liver, *DG* diaphragm

Concerning the ORF2 sequenced fragments, 37/55 (67.27%–95% CI: 54.87–79.67%) of them (from liver samples) were identical and registered on the GenBank with the accession number MN2020210201, and 18/55 (32.72%–95% CI: 20.32–45.12%) were identical (MN2020210208). Positive diaphragm sequences resulted identical (MN2020210212). The sequences, obtained from positive animals in the liver and muscle tissues, presented high nucleotide similarities (nt. 98.00%), as observed from ORF1 fragments (MN20202103 and MN20202104).

Basing on the nucleotide similarities with the discovered sequences from the human species (*Homo sapiens*), the obtained ones, in this study belonging to the ORF1 fragments, were 91.00% identical to the MF346772.1 that was discovered from human stools in Switzerland. The ORF2 ones were also 95.00% identical to HEV3 with accession number MH427093.1 discovered from human feces in Thailand.

## Discussion

From an ecological point of view, HEV biological characteristics are the evolutive result of fascinating and continuous interactions between virus, host species, and environment (Aspinall et al., 2017). Interpreting the environment from a macro (biotope, anthropological & cultural features) and micro (virus & hepatocyte biomolecular interactions) point of view, the obtained scientific findings, discovered in this epidemiological investigation, can be projected into different ecological levels, understanding the essential basis of the virus-host species ecology (Di Cola et al., 2021). In Central Italy, traditional foodstuffs as primary and processed products (salami, sausages, etc.) are obtained from wild boar liver and muscle tissues. Every year, many HEV infections are registered in Marche (30.4%), Abruzzo (29.1%), and Toscana (17%) regions (SEIEVA, 2021). In these areas, there is a rooted wild boar hunting tradition. The risk of infection for humans has been observed by serological investigations, as reported by Raji et al. (2022) reporting high seroprevalence’s titers in hunters and by Spada et al. (2022) from blood donors. Translating this concept to the HEV population dynamics, the most diffused genotype in Europe is represented by HEV3 (Salines et al., 2017).

In agreement with the previous studies, performed in many Italian regions, the present scientific investigation discovered the subtype 3c. The HEV3c circulation, in the southern Marche region, was also observed by previous studies (Ferri et al., 2022a, 2022b) performed in the same screened province (Ascoli Piceno), and in the adjacent Southern Central Italian areas (Aprea et al., 2018; Di Pasquale et al., 2019; Montagnaro et al., 2015).

The 10.87% (95% CI: 8.16–13.58%) of liver samples resulted positive for HEV RNA detection. The obtained result was higher than the two previous studies performed in the Ascoli Piceno province. More in detail, the two mentioned molecular screenings reported 5.12% of positive livers, discovered in 2019, (Ferri et al., 2022a), and 5.45% in the hunting season 2020 (Ferri et al., 2022b). These differences found explanation from the increasing of wild boar populations in Central Italy, as denounced by the SEIEVA report (Fanelli et al., 2021). Concerning diaphragm samples, as reported in the Table 3: Results section, 14/506 (2.76%–95% CI: 1.34–4.18%) were positive. Similarly, to the data observed from liver tissue, the obtained positive diaphragmatic prevalence resulted also higher than the previous investigation (in 2020) 1.35% which was also performed in the same geographical area (Ascoli Piceno province) (Ferri et al., 2022b). The obtained data can be justified by the possible viremic *status*. Furthermore, it is possible to exclude any cross-contamination due to the anatomic contiguity or during samples collection. This last affirmation is supported by the application of a specific sampling method, as reported by Dzierzon et al. (2021), which provide all procedures to avoid cross-contamination from organ serosa to the respective parenchyma during the section steps. Diaphragm muscle was included because it is largely consumed (alone or mixed to other muscular groups) as fresh meat or included in processed pork products in Central Italy (SEIEVA, 2021). However, it is not possible to exclude that any liver fragments can be included during diaphragm collection (related to many organ manipulations performed by hunters or slaughterhouse personnel). For sanitary purposes, in the European scenario, the Dutch Meat Products Association highly recommended banning pork diaphragms to be introduced as ingredient. Indeed, Tulen et al. (2019) discovered a consistent reduction in the HEV RNA positivity of fresh and processed pork meat products (60–70% of decreasing) if producers avoided using the above-mentioned muscular anatomical part. These criteria could find reasonable applicability in the screened province to reduce the viral circulation and the related high seroprevalence titers. Comparing the obtained data with other Italian regions, it resulted in agreement with their prevalence values (from liver tissue), observed in Abruzzo (13.7%), Lazio (12.1%) (Aprea et al., 2018) regions, but lower than the 31.5% and 23.8% reported in Emilia-Romagna one, reported by Bonardi et al. (2020) and Arnaboldi et al. (2021). These different prevalence values could be the expression of a HEV3 environmental resistance and wild boar behavioral peculiarities representing one of the crucial aspects of HEV diffusion among domestic and wild animal species (Subissi et al., 2019).

Concerning positive animal gender, female subjects resulted more frequently positive than male ones; indeed, between these two groups, the statistical analysis revealed a statistically difference presenting a final *p*-value: < 0.002. More in detail, adults were statistical majorly positive than puberal ones. In the puberal category, more positive female subjects were more than male ones, in agreement with previous studies (Arnaboldi et al., 2021; Bonardi et al., 2020). This last finding supports the scientific hypothesis formulated by many authors that focused on the potential role of sexual hormones peaks (mainly associated with estrogens synthesis in the puberty phase) which may determine the highest host receptivity to the viral infection (Arnaboldi et al., 2021; Oechslin et al., 2020). Therefore, HEV developed its invasive strategies encrypting the host’s endocrine signals, identifying puberty as the “*trigger*” event that induces a dynamic waterfall-series of molecular mechanisms favorable to the cell invasion, as affirmed by Oechslin et al., 2020. From a perspective based on the "*virus-cell molecular interactions*,” enterocytes and their syndecan 1 represent the first cellular anatomical molecular sites and frontiers for HEV invasion and adhesion. Following the biological meaning of syndecan as a “*key-role site*” molecule in viral adhesion and invasion, observing the syndecane expression levels in different tissues and organs, mainly expressed by hepatocytes and skeletal myocytes (Oechslin et al., 2020; Pisconti et al., 2012). The hepatocytes’ syndecan high expression level makes this cytotype one of the most receptive to HEV adhesion (Oechslin et al., 2020). These considerations create the optimal microenvironment for viral nuclear replication and represent one of the biological discussions explaining the higher liver sample positivity concerning that observed in diaphragm ones (See Results section) (Pisconti et al., 2012).

From this epidemiological investigation, the gender variable, related to the sexual hormones levels, poses the basis for further studies which will focus on the increased host receptiveness to the viral pathogens, as previously hypothesized by Neal et al. (2012). This endocrine and myocellular glycoproteome microenvironment may represent the molecular basis explaining the high percentage of males in HEV RNA positive diaphragm samples 10/14 or the 71.43% (95% CI: 47.77–95.09%) compared to those of females 4/14 or the 28.57% (95% CI: 4.91–52.23%) (as described in the Results section). These last considerations pose the basis for further investigations giving reasonable scientific explication concerning the hormone-dependence of viral multiplication.

## Conclusion

From humans to the wild boars, from forest to the cells, HEV reveals how virus, host, and environment are three “*main actors*” strictly interconnected and adapted to the anthropological and cultural features of a society, all acting in a dynamic macroenvironment. In this riveting pathogenetic story, each “*actor*” shows its own strategies evolved in million years, guided by the evolution of an astonishing biochemical intelligence incessantly operating in the molecular-scale microenvironment. It is mandatory to affirm that the discovered sequences were short genome fragments, and for this reason, the amplified determinants led to a preliminary scientific consideration highlighting HEV-3c as the main identified genotype. The interaction between virus and host animal species, humans included, in the macro and microenvironment scenarios determines pathogenetically and epidemiological features of HEV, revealing in this riveting story the evolutive drawing of life.
